# The Impact of Egg Nutrient Composition and Its Consumption on Cholesterol Homeostasis

**DOI:** 10.1155/2018/6303810

**Published:** 2018-08-23

**Authors:** Heqian Kuang, Fang Yang, Yan Zhang, Tiannan Wang, Guoxun Chen

**Affiliations:** ^1^Department of Nutrition, University of Tennessee at Knoxville, Knoxville, Tennessee, USA; ^2^School of Laboratory Medicine, Hubei University of Chinese Medicine, Wuhan, Hubei, China

## Abstract

Nutrient deficiencies and excess are involved in many aspects of human health. As a source of essential nutrients, eggs have been used worldwide to support the nutritional needs of human societies. On the other hand, eggs also contain a significant amount of cholesterol, a lipid molecule that has been associated with the development of cardiovascular diseases. Whether the increase of egg consumption will lead to elevated cholesterol absorption and disruption of cholesterol homeostasis has been a concern of debate for a while. Cholesterol homeostasis is regulated through its dietary intake, endogenous biosynthesis, utilization, and excretion. Recently, some research interests have been paid to the effects of egg consumption on cholesterol homeostasis through the intestinal cholesterol absorption. Nutrient components in eggs such as phospholipids may contribute to this process. The goals of this review are to summarize the recent progress in this area and to discuss some potential benefits of egg consumption.

## 1. Introduction

A homeostatic cholesterol regulation system exists in the human body. The loss of cholesterol homeostasis results in abnormal cholesterol metabolism such as hypercholesterolemia, which is associated with the development of cardiovascular diseases (CVD) including atherosclerosis, coronary heart disease, and stroke. CVD is one of the leading causes of death globally, which resulted in 17.9 million deaths (32.1%) in 2015 and was increased from 12.3 million (25.8%) in 1990 [1]. According to results of epidemiology studies, high plasma cholesterol level, especially high level of low-density lipoprotein (LDL), is directly associated with CVD, while the leve of high-density lipoprotein (HDL) is negatively related to CVD risk [2]. The relationship between blood cholesterol and heart disease was firstly confirmed by the Framingham Heart Study [3], and the hypothesis that dietary cholesterol is also associated with heart diseases was proposed [4]. The hypothesis was supported by animal study in rabbits in 1913 [5] and observational studies in 1980s [6, 7], demonstrating a link between dietary cholesterol and risk of heart diseases.

In 1968, the American Heart Association (AHA) recommended that the dietary cholesterol consumption should be no more than 300 mg/day and emphasized no more than 3 egg yolks should be consumed per week [8]. The average intake of dietary cholesterol in US adults ranges from 200 to 350 mg/day due to sex and age differences [9]. Daily intake of eggs and egg products in US children and adults accounts for 25% of daily total cholesterol intake [10, 11]. Some researchers recommended that food which is rich in cholesterol, such as eggs, should be limited in an attempt to lower the plasma cholesterol level [3]. A series of “low cholesterol” and “cholesterol free” food advertised on media. The “evil” of cholesterol and eggs has been announced to publics constantly, which still exists nowadays.

The following studies showed contradictory results. Shekelle (1989) and Kushi (1985) found dietary cholesterol was correlated to CVD risks, but they failed to take many confounding variables into account, such as genes, hormones, other dietary components, lifestyle, and the nutritional status of the subjects [6, 7]. On the other hand, later epidemiological studies and systematic reviews reported a marginal correlation between dietary cholesterol and/or egg intake and CVD incidence in the general population [12–23]. With increasing evidences of weak correlation, AHA eliminated the restriction of egg consumption in 2002 and the dietary restriction of cholesterol for healthy population in 2013 [24]. Furthermore the US Dietary Guidelines Advisory Committee (DGAC) also eliminated this restriction from the latest version of dietary guideline in 2015 [25].

After being debated for half a century, dietary cholesterol and egg intake finally got rid of the nature of “evil.” Moreover, recent studies reported that multiple bioactive components in eggs have potential benefits to overall health through the lifespan, such as supplying micronutrients, antioxidants, antimicrobials, and reducing risk of cancer and hypertension [26]. This brings the nutrient value of eggs back to a hot spot. This review aims to summarize recent research progress on the composition and nutritional value of egg components, cholesterol homeostasis, the relationship between dietary egg intake and blood cholesterol, and the effects of different egg components.

## 2. Composition and Nutritional Value of Egg Components

Eggs, an inexpensive but highly nutritious food, provide balanced nutrients that impact human health [25, 26]. Eggs contain ample essential proteins, fats, vitamins, minerals, and bioactive compounds, and their compositions and net amount could be influenced by strain, age, hen diet, and environmental conditions. The nutrients to energy density ratio of one egg is high with many essential nutrients as shown in Table 1 [27]. A medium-sized boiled egg (50 g) contains 78 kcal energy, 6.29 g protein, 0.56 g carbohydrate, and 5.3 g total fat, of which 1.6 g is saturated, 2.0 g is monounsaturated, 0.7g is polyunsaturated, and 186 mg is cholesterol. As for micronutrients, egg contains a variety of minerals (calcium, iron, magnesium, phosphorus, potassium, sodium, and Zinc) and most vitamins (thiamin, riboflavin, niacin, vitamin B_6_, folate, vitamin B_12_, vitamin A, vitamin E, vitamin D, and vitamin K) except for vitamin C. Some of these nutrients, such as zinc, vitamin A, vitamin D, and vitamin E, may not be enough in a western diet.

### 2.1. Egg Proteins

For humans, eggs are one of the best sources of high quality protein, only inferior to breast milk. Egg proteins have been proved to possess antioxidants, such as phosvitin which contains large amount of phosphoserines, ovotransferrin that can chelate with Fe^3+^, and ovalbumin that can covalently bind to polysaccharide to enhance its antioxidant activity [28, 29]. These proteins can inhibit lipid oxidation by binding to metal or scavenging free radical. Eggs may be used as a potential natural source of antioxidant, which can be further used in food or cosmetics industry. The antioxidant function of eggs could prevent humans from a large number of degenerative processes, such as CVD occurring [30]. Besides, egg protein, especially egg yolk protein, has a significantly greater satiety effect compared with other protein sources [31, 32]. Some research results have shown that there was reduced energy intake after the breakfast with an egg and a bagel [33], larger weight loss after 8 weeks with the breakfast containing an egg and a bagel as part of a hypocaloric diet [34], and significant change in satiety hormones after an egg in the breakfast [35]. Proteins in egg yolk can be classified into apolipoproteins, phosvitin, egg yolk globulin, and riboflavin binding protein [36].

### 2.2. Egg Lipoproteins

**Figure 1:**
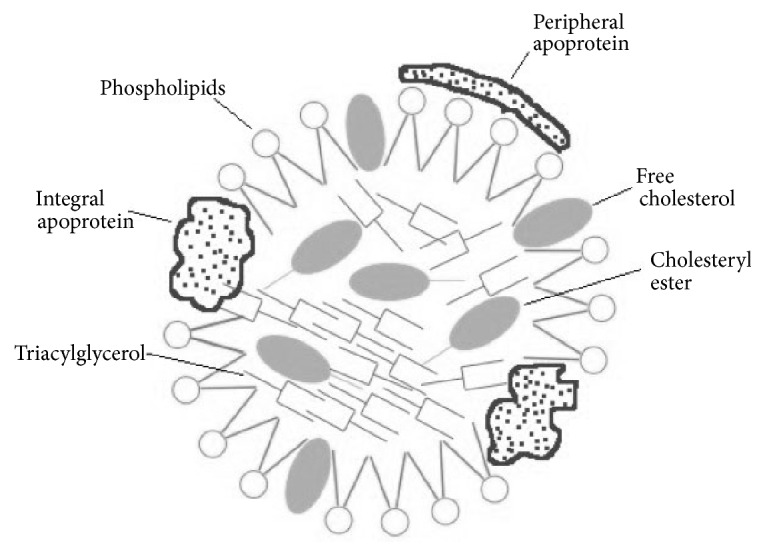
**The structure of egg yolk lipoprotein**. The outer layer of egg yolk lipoprotein is mainly composed of phospholipids, with free cholesterol scattered and integral and peripheral apolipoprotein attached. The hydrophobic triacylglycerol and cholesteryl ester exist in core.

Egg lipoproteins include LDL and HDL. The LDL in the yolk accounts for about 2/3 of the dry matter of the egg yolk which can be divided into the soluble part and whey part [37]. The density of LDL is 0.982 g/ml. LDL particles are spherical and the average diameter is between 35 and 40 nm. As shown in Figure 1, the core of the particles is made of triglycerides (TG) and cholesterol esters (CE) that are surrounded by apolipoproteins, phospholipids, and cholesterol [38]. Montserret et al. demonstrated that LDL can disperse at the oil-water interface, with phospholipids and apolipoproteins attached to the surface of the nucleus, and the central core combines with oil droplets [39]. There are six major apolipoproteins: A, B, C, D, E, and H [38]. Apolipoprotein with molecular weight of 15 kDa is the most active form on the surface of microparticles. Apolipoproteins contain higher proportion of amphipathic *α*-helices after extraction. Therefore, apolipoproteins can be adsorbed effectively at the oil-water interface [38]. More than 95% of the yolk cholesterol are stored in LDL, more than 90% of which present in free (nonesterified) form [40]. Free cholesterol plays an important role in maintaining the structure of lipoproteins. They are packed between adjacent phospholipid molecules to maintain the stability of the oil/water interface [40]. Almost 98% of sterols in the egg yolk are cholesterol, the rest of which are a small amount of phytosterols such as beta-sitosterol, methyl cholestereol, and sorbitol mycotoxins [41].

### 2.3. Egg Lipids

In addition to protein, eggs also contain a large number of active lipid components, such as unsaturated fatty acids, phospholipids, choline, and carotenoids. As shown in Table 1, monounsaturated fatty acids (MUFAs) and polyunsaturated fatty acids (PUFAs) are, respectively, 2.0 g and 0.7 g, and content of saturated fatty acids is 1.6 g in one medium-size egg. Phospholipids account for approximately 10% of the wet weight of egg yolk [42], which mainly includes phosphatidylcholine (PC), phosphatidylethanolamine (PE), lysophosphatidylcholine (lysoPC), sphingomyelin (SM), and some neutral lipids in minor quantities. Dietary phospholipids, a potential source of bioactive lipids, may have broad effects on cholesterol metabolism, HDL functions, and inflammation [43]. Yang et al. reported that dietary PUFAs, PC, and SM significantly inhibit the uptake of cholesterol in Caco-2 monolayer, which may have potential therapeutic effect on reducing cholesterol absorption as functional food ingredients [44, 45]. As a component of lecithin, choline also exists in egg in a larger amount [46]. Choline may play a particularly useful role in fetal and neonatal brain development, as inadequate choline intake during pregnancy increases neural tube defects risk of infant [47, 48]. Other functional components from egg are the carotenoids, which are natural pigments in hen egg yolks and account for less than 1% of yolk lipids [28, 49]. The two major carotenoids in eggs are carotene and xanthophylls (lutein, cryptoxanthin, and zeaxanthin) which are highly bioavailable in egg yolk and are associated with the reduced risk of age-related macular degeneration and cataracts, cancer, and carotid artery atherosclerosis [28, 49, 50].

## 3. Regulation of Cholesterol Absorption

Cholesterol homeostasis depends mainly on the balance among cholesterol absorption in the intestine, endogenous cholesterol synthesis, and utilization for the synthesis of bile acids and steroids. High blood cholesterol level is an important risk factor for CVD. Therefore, a large number of studies focused on investigating the key genetic, physicochemical, and biochemical indicators of intestinal cholesterol absorption in the small intestine.

### 3.1. Physiological Process of Cholesterol in the Small Intestine

There are three sources of cholesterol in the small intestine: diet, bile, and shedding of intestinal epithelial cells [51]. The daily cholesterol intake in a typical Western diet is about 300-500 mg/day. Bile contributes to 800-1200 mg of cholesterol per day in the small intestine lumen [51]. Although the entire small intestine can absorb cholesterol, the main sites of absorption are the duodenum and the proximal jejunum. TGs are hydrolyzed by pancreatic lipase, phospholipids are hydrolyzed by pancreatic phospholipase, and CEs are hydrolyzed by carboxylester lipase. The digestive products are monoacylglycerides, small amount of diacylglycerols, lysophospholipids, free cholesterol, and free fatty acids. These products can form micelles with bile acids, cross the unstirred water layer on the side of the brush boarder, and absorbed into enterocytes by passive diffusing or mediated transporting [51]. Hydrophilic bile acids can reduce the absorption of cholesterol in the small intestine by decreasing the solubility of cholesterol on the out-shell of micelles [52]. Compared to the cholesterol, sterol and plant phytosterols are easier to be incorporated into micelles, which leads to the reduction of cholesterol absorption efficiency in the small intestine [52]. In the enterocytes, TGs, CEs, and phospholipids are generated again and packed into chylomicrons for distribution in the lymphatic circulation and then the blood [51]. Upon the hydrolysis by lipoprotein lipase, chylomicrons eventually become chylomicron remnants, which are taken into the liver. The lipids in chylomicron remnants are reassembled into very-low-density lipoproteins [51].

Cholesterol is specifically absorbed by Niemann-Pick C1-Like 1 (NPC1L1), which is a newly discovered transporter, and excreted by ATP-binding cassette (ABC) G member 5 (G5) and G8, which can also speed up the absorption of cholesterol, on the apical side of small intestine epithelium membrane [53]. Ezetimibe reduces the absorption of cholesterol in the small intestine by inhibiting the activity of NPC1L1 protein [52]. Nearly half of cholesterol molecules are transported to the endoplasmic reticulum (ER) after entering the epithelium and reesterified into CEs by Acyl-coenzyme A:cholesterol acyltransferases (ACAT) [53]. These CEs are transported to the Golgi apparatus and then loaded unto chylomicrons via microsomal triglyceride transfer protein (MTP). Free cholesterol also participates in forming the shell of chylomicron precursors [53]. Part of cholesterol and bile acids can escape the absorption in the small intestine and are excreted in the stool as neutral sterols and acidic sterols, which is the major elimination route of sterols from the body [52]. The exact molecular mechanisms of intestinal cholesterol absorption are still under investigation.

### 3.2. Factors Affecting the Efficiency of Intestinal Cholesterol Absorption

The cholesterol absorption efficiency varies among animal species and human subjects.  Table 2 summarizes the key factors affecting cholesterol absorption including dietary factors, pharmacological influence, bile acid factors, genetic factors, and intestinal lumen factors. Intestinal absorption efficiency can also be affected by the factors that influence cholesterol transport from the small intestine to the lymphatic system. Changes in these factors can help to explain the differences in the absorption efficiency of cholesterol in the small intestine within individuals and species.

Fang et al. evaluated the effects of six fatty acids, palmitic acid, oleic acid, linoleic acid, arachidonic acid, eicosapentaenoic acid (EPA), and docosahexaenoic acid (DHA), on cholesterol uptake and transport in human enterocytes Caco-2 cells and on the expression levels of NPC1L1 and others proteins involved in cholesterol absorption. The results showed that EPA and DHA dose-dependently inhibited cholesterol uptake and transport in Caco-2 monolayer, which might be caused by downregulating NPC1L1 level [45].

When* Abcb4*, an ABC transporter gene encoding hepatic microtubule flinty flippase, was knocked out in mice, the secretion of bile acid and phospholipids was inhibited. In homozygous and heterozygous* Abcb4*-deficient mice, the output of bile acid and phospholipids is important for maintaining normal cholesterol absorption in the small intestine [54]. In cholesterol 7*α*-hydroxylase gene (*Cyp7a1*) knockout mice, bile acid output and bile acid pool size decreased significantly, as a result of limited absorption of cholesterol due to the lack of bile acids [55].* Cyp27a1* gene encodes one of the key enzymes for bile acid synthesis pathways [56]. Therefore, the* Cyp27a1* gene-knockout mice have decreased bile acid pool size. The intestinal absorption of cholesterol decreased from 54% to 4%, and feces bile acid excretion increased 2.5-fold in* Cyp27a1* knockout mice, while feeding them a bile acid-rich diet reversed the impairment of intestinal cholesterol absorption [56]. These findings demonstrate that both the size of bile acid pool and the output of bile salts play important roles in the intestinal absorption of cholesterol [57].

Human and animal experimental studies have found that short transit time in the small intestine decreases absorption of cholesterol [58]. Cholecystokinin-1 receptor gene (*Cck1*) knockout mice showed a higher cholesterol intestinal absorption efficiency, resulting in the excessive secretion of bile cholesterol and the formation of cholesterol stones [59]. However, in the cholesterol congenital fatigue mice, the intestinal transit time is as important as the length and weight of the mouse small intestine [60].

The increase of age will lead to significant increase of intestinal cholesterol absorption efficiency in rodents [61, 62]. Sexual differences will also lead to different cholesterol absorption efficiency probably due to female hormones. The secretion of bile lipids (bile salts, phospholipids, and cholesterol), the concentration of cholesterol in bile, the size of the bile pool, and the hydrophobicity of the bile salt pool increase significantly with age. The mixture of bile factors has a significant impact on the absorption of cholesterol efficiency in the small intestine with the increase of age. In addition, estrogen can significantly increase the bile flow rate and the output of bile salts, cholesterol, and phospholipids [63, 64].

### 3.3. Methods of Measuring Cholesterol Absorption Efficiency in the Small Intestine

Accurate measure of the small intestinal cholesterol absorption is essential for the determination of the dynamic cholesterol homeostasis. In healthy congenital fatigue mice, the range of cholesterol absorption efficiency varies widely from 20% to 90% based on the stool double isotope proportional labeling method [57]. There are direct and indirect methods to detect intestinal absorption rate of cholesterol in mice. The direct method is lymphatic intubation which detects cholesterol transport from the small intestine to the mesenteric and thoracic lymphatic ducts. This requires special lymphatic and duodenal cannulas to measure perfusion of cholesterol [97, 98]. The most common method is the stool double isotope ratio labeling method. Radio-labeled cholesterol and labeled nonabsorbed phytosterols used as markers were fed to the subjects [99, 100]. This method relies mainly on the accurate detection of excretion and ratio of two isotopes in the stool [57]. The simplest method for testing of the intestinal cholesterol absorption is stool and plasma dual plasma isotope labeling method. Both [^3^H] and [^14^C]-labeled cholesterol are injected intragastrically and intravenously. The isotope ratio of plasma cholesterol is then periodically measured. Intravenous injection is assumed as the 100% absorption reference [100]. Sterol balance method is based on measuring the difference between cholesterol intake and excretion when testing the absorption of exogenous sterol. The detection of external excretion of neutral steroid is the key point in this method [57].

Under high cholesterol intake condition, stool and plasma dual isotope labeling method is not suitable to test the intestinal cholesterol absorption [57]. When the dietary cholesterol intake increases, intestinal absorption of cholesterol decreases, while the expression of steroid efflux transporters ABCG5 and ABCG8 are upregulated. In such cases, sterol balance and the lymphatic intubation methods are good ways, which detect not only the percentage but also the amount of cholesterol absorption from the lumen of small intestine [57]. Applying labeled sitosterol and sitostanol as reference markers may influence the accuracy of intestinal cholesterol absorption. Therefore, in animal and human studies, sterol balance method is an ideal method to test the intestinal cholesterol absorption.

## 4. Regulation of Cholesterol Metabolism

### 4.1. Cholesterol Biosynthesis in the Body

**Figure 2:**
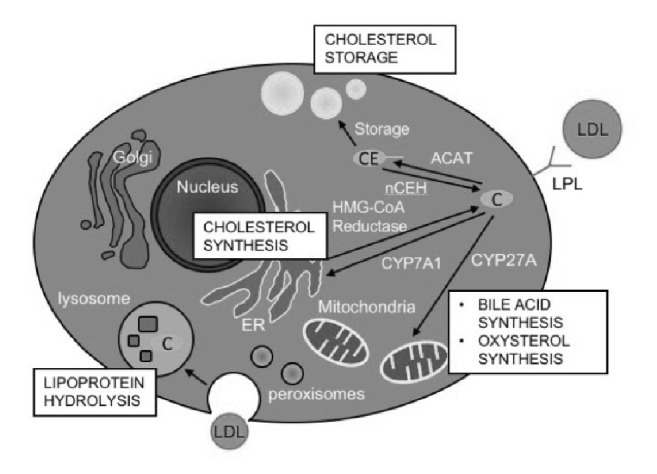
**The cellular cholesterol partition and key enzymes of cholesterol metabolism**. The cholesterol (C) from low-density lipoprotein (LDL) could be uptaken by liver cell via two ways, the endocytosis and hydrolyze LDL via lysosome or digest and uptake via lipoprotein lipase on the cell membrane. Cholesterol could be esterified into cholesteryl esters (CE) for storage in liver or be used for synthesize bile acid or oxysterol via cholesterol 7 alpha-hydroxylase (CYP7A1) on endoplasmic reticulum (ER) or via sterol 27-hydroxylase (CYP27A) in mitochondria. Cholesterol could also be synthesized de novo via enzyme on ER.

Cholesterol biosynthesis is a complicated 37-step process. These intracellular steps occur in the cytosol and ER and use acetyl CoA to make a 30-carbon linear molecule, squalene, which is further cyclized and modified into the 27-carbon cholesterol [101, 102] (Figure 2). In the cholesterol synthetic pathway, the first cyclized sterol product derived from squalene is lanosterol which is catalyzed by oxidosqualene cyclase [101, 103]. The remaining 19 steps include methyl oxidation, alkene shifting, and ketone reduction [101, 103]. From the acetyl CoA to cholesterol, the rate-limiting step of cholesterol synthesis is catalyzed by hydroxymethylglutaryl-CoA (HMG-CoA) reductase which is tightly regulated [101, 103].

Majority of the enzymes catalyzing the first phase from acetyl CoA to lanosterol are membrane proteins located in the ER inner membrane including HMG-CoA reductase [104]. Other enzymes in this phase also locate in peroxisome, such as mevalonate kinase, or in cytosol, such as HMG-CoA synthetase [104, 105]. Enzymes converting lanosterol into cholesterol mainly locate in ER and its extension parts, nuclear membrane, or lipid droplets [102].

### 4.2. Cholesterol Homeostasis and Regulation

**Figure 3:**
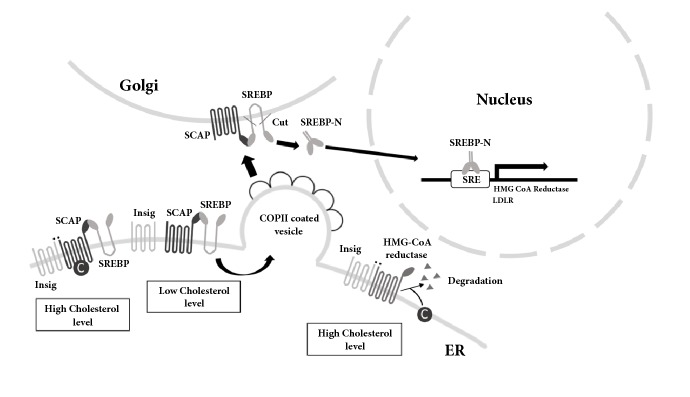
**Regulation of cholesterol homeostasis via SREBP and HMG-CoA reductase**. When the cholesterol (C) or oxysterol level is high, they can bind to sterol sensing domain (SSD) of the sterol regulatory element binding protein (SREBP) cleavage activating protein (SCAP) or HMG-CoA reductase. In this case, SREBP will be stabilized on ER to be inactivated, and HMG-CoA reductase will be degraded therefore cholesterol synthesis will be inhibited. The low cholesterol level causes release of Insig and allows the SCAP/SREBP to enter the COPII coated vesicle. The vesicle will move to Golgi and the SREBP will be cleaved into the activated form. The mature SREBPs enter into nucleus to bind to sterol regulatory elements (SREs) to induce the downstream genes expression.

The intracellular cholesterol level is monitored and regulated by the proteins on ER membrane. SREBP and LXR are the important transcriptional factors involved in cholesterol homeostasis [104–106]. SREBPs increase the transcription of genes encoding proteins in the cholesterol biosynthetic pathway, such as HMG-CoA reductase, while LXRs activate the expression of genes encoding proteins involved in cholesterol metabolism (Figure 3) [106–108].

The cholesterol level in ER is sensed by a sterol sensing domain (SSD) composed of five membrane-spanning *α*-helices, which exist in many cholesterol sensing proteins [109]. When the cholesterol or lanosterol level is high in the cell, the SSDs of HMG-CoA reductase and SREBP cleavage activating protein (SCAP) sense the sterol and cause those proteins to bind to Insig in the ER membrane [106]. Insig binding to the HMG-CoA reductase causes it to be degraded, which reduces the cholesterol synthesis rate [106]. On the other hand, the binding of Insig to SCAP entrenches the SREBPs on the ER, which decreases the transcription of genes for cholesterol synthesis. When the intracellular sterol level is low, the SSDs of HMG-CoA reductase and SCAP dissociate and release the Insig on ER. As a result, the reductase will keep working and produce cholesterol, and SCAP-SREBP complex will be associated with a coat protein complex II (COPII) vesicle and be transported to the Golgi where proteases cut SREBPs and release the active SREBPs. The active SREBPs enter the nucleus, bind to the SREBP regulatory element (SRE) and stimulate the transcription of genes such as HMG-CoA reductase [101, 109].

LXRs regulate gene expressions in diverse processes of metabolism [101, 109]. LXR and retinoid X receptor (RXR) bind to their ligands and form a heterodimer to bind to the promoter of their targeted genes to regulate their expression, including genes for sterol metabolism [101, 108]. When sterol metabolites, which are ligands of LXR, levels are high, they bind to LXR to regulate genes involved in cholesterol metabolism and excretion, such as Cyp7a1 which catalyzes the rate-limiting step of hepatic bile synthesis from cholesterol and ABCG5/G8 which are cholesterol efflux pumps. As a result, the cholesterol will be used or excreted out of the cell to decrease its intracellular concentration. However, when those sterol metabolites levels are low, the lack of ligands for the activation of LXRs will lead to the reduction of those genes' expression [101, 108].

Nearly 75% of serum cholesterol in humans is derived from cholesterol biosynthesis while the rest is derived from the diet. About 850 mg cholesterol is synthesized by an average 70-kilogram adult per day. If the person consumes 400 mg/day cholesterol from diet and absorbs 60% [110], that means only 22% of cholesterol handled in the body comes from the diet (240 mg from the diet out of a total of 1090 mg). Lin and Connor reported that consuming diets with high cholesterol could cause feedback inhibition of cholesterol biosynthesis and increase of bile acid excretion [111, 112]. Such tiny changes with dietary cholesterol intake and the tight feedback control can limit its effect on cholesterol homeostasis in the body. Therefore, a marginal change in serum cholesterol for most healthy people occurs in response to the dietary cholesterol level change.

### 4.3. Cholesterol Esterification and Hydrolysis of CE

The 3′-OH group of cholesterol can be esterified into CE which can be stored in cytosolic lipid droplet or used to form lipoproteins. ACAT catalyzes this esterification reaction and is widely expressed in different types of animal cells [113, 114]. There are two isoforms of ACAT. ACAT1 catalyzes the esterification and storage of CE in the lipid droplets in macrophages, whereas ACAT2 catalyzes the esterification and incorporation of CE into lipoproteins, such as chylomicrons in the intestinal epithelial cell and very-low-density lipoprotein in the liver. ACAT is an integral membrane protein which is mainly found on ER membrane [113, 114], but, in macrophages, it is also found near the trans-Golgi network and endocytic recycling compartment [115]. The esterification of cholesterol and hydrolysis of CE are in a continuous intracellular cycle. When the cholesterol level is high, ACAT is activated and esterifies cholesterol into CE for storage, while when the cholesterol level is low, neutral cholesteryl ester hydrolases (nCEHs) catalyze the reverse reaction to release cholesterol into cells (Figure 2) [116]. nCEHs are also widely expressed in tissues. The regulation of nCEHs activity is not well known, and only the nCEH in the liver is thought to be regulated by the cholesterol flux [116, 117].

### 4.4. Cholesterol Metabolites

Cholesterol can be converted into bile acids in the liver or secreted into bile as free cholesterol to be excreted in feces [116, 118]. Therefore, the cholesterol in extrahepatic tissues needs to be taken into circulation and transported back to the liver for secretion [116, 118]. There are two pathways for the conversion of cholesterol into bile acids: the classic pathway and the alternative pathway [119]. The rate-limiting enzyme for classic pathway is CYP7A1 which locates in ER. The enzyme for the alternative pathway is sterol 27-hydroxylase (CYP27A) which locates in mitochondria (Figure 2) [116, 120]. Classic pathway exists specifically in the liver, while the alternative pathway is found in almost all tissues and has been considered for oxysterols production in extrahepatic tissues [116]. Oxysterols, derived from cholesterol, can serve as ligands for the activation of LXRs [108, 116]. Because of their hydrophilic feature, oxysterols represent another way to move cholesterol back to liver from other tissues, which is different from the way via lipoproteins [113, 116]. Steroid hormone is another class of sterol which is produced from cholesterol and plays an important role in the growth and development of animals. There are five classes of steroid hormones in mammals: glucocorticoids, mineralocorticoids, progestins, and androgens [121]. Another functional steroid, neurosteroid, function as neurotransmitters in the brain. The functions of neurosteroids include regulation of axons' and dendrites' growth, neuroprotection, and myelinization [122].

## 5. The Relationship between Egg and Cholesterol Homeostasis

### 5.1. Effect of Egg Intake on Lipoprotein Metabolism

The potential influence of egg consumption on CVD probably could be mediated by the influence of egg consumption on lipoprotein level, lipoprotein particle feature, HDL metabolism, and functionality. Studies have shown that although consuming eggs often results in the increases of both serum LDL and HDL levels, the LDL/HDL ratio is unchanged (Table 3). Therefore, the adverse effect of LDL on CVD might be counteracted by the beneficial effect of HDL. It has been shown that many other lipoprotein features which correlate to CVD risks can also be changed by egg consumption [20, 92, 93, 123–126]. The cholesterol needed by peripheral tissues including blood vessels can be transported by LDL, and when LDL level is too high, it accumulates in the artery wall, causing inflammation and oxidation [127]. This leads to uptake of cholesterol by the foam cells, resulting in endothelial damage and the occurrence and development of atherosclerosis. HDL removes the cholesterol from the foam cells to inhibit LDL oxidation, attenuate inflammation, and reduce the transport of cholesterol to prevent atherosclerosis, and HDL also has the characteristics of being antithrombotic [127].

The serum lipid responses to dietary cholesterol were tested by Berger et al. [128] across 19 intervention trials. Serum LDL-Cholesterol (LDL-C) (6.7 mg/dL net change) and HDL-C (3.2 mg/dL net change) are greatly increased by dietary cholesterol intake, mostly from eggs, with a marginal increase in the LDL-C/HDL-C ratio (0.17 net change) [128]. The ratio of LDL-C/HDL-C can be used to estimate the likelihood of forming plaque in blood vessels, and therefore it can be used as an indicator of CVD risk [129]. LDL-C/HDL-C ratio <2.5 is considered to be the cut-point of personal lipoprotein recommendations, and there is evidence that, in some people above this level, the risk of cardiovascular events is higher [129, 130]. Other studies in children and adults with normal cholesterol levels also reported the significant increase of LDL-C and HDL-C with 2-4 eggs' intake per day comparing to without egg yolk, while the LDL-C/HDL-C ratio did not change [92, 123, 124, 131]. Although Herron reported an increase of LDL-C/HDL-C ratio consuming 3 eggs per day for 30 days in healthy men who were classified as hyperresponders (increase in total cholesterol of ≥0.06 mmol/L for each additional 100 mg of dietary cholesterol consumed), the average ratio (2.33±0.80) was still in the normal range of <2.5 [126]. In adults with high cholesterol, eating two eggs per day could lead to higher HDL-C without a change in LDL-C, while both the HDL-C and LDL-C increased in adults with hyperlipidemia (elevated serum cholesterol and triglyceride) [132]. In the elderly who took statins, two or four eggs per day did not significantly increase LDL-C, while HDL-C was increased with both doses of eggs [133]. And consumption of eggs might have contributed to the conversion of LDL from pattern B particles into pattern A in the human body, and the pattern A LDL particles have large size and are easy to float, which reduces their potential to stimulate the development of atherosclerosis [123].

Although LDL-C and HDL-C levels are related to CVD risk, some features of lipoproteins may also affect disease risk, such as particle characteristics, particle size, and composition. For example, higher plasma concentrations of large HDL subclass particles are strongly positively correlated to CVD risk, while the correlations between the lower level of small HDL particles and the risk of CVD are weak [134, 135]. However, higher plasma concentrations of small LDL particles are highly related to CVD development, explained by its higher oxidation susceptibility than larger LDL particles [135, 136]. Oxidized LDL is a major driver of plaque formation, which results in atherosclerosis and CVD [137].

HDL is believed to play a protective role in atherosclerosis through its role in reverse cholesterol transport (RCT), as well as its antioxidant and anti-inflammatory activities [138]. Dietary cholesterol feeding in mice has been proved to induce RCT by compensatory induction through HDL related pathways [139]. With the intake of cholesterol and/or eggs in diet, it is not clear whether the increase of HDL-C level and particle size would result in increasing of RCT in human. Several drug trials have failed to show the benefits of improving HDL-C on CVD [140, 141]. However, studies have shown that dietary cholesterol and/or eggs may affect the function of HDL other than modifying HDL-C level. Dietary cholesterol intake independently predicts the activity of serum paraoxonase-1 (PON1) arylesterase in a human study [142]. PON1 is a lipolactonase carried by HDL, which may protect atherosclerosis by preventing lipoprotein oxidation, inhibiting macrophage inflammation, and enhancing cellular cholesterol outflow mediated by HDL [143]. Interestingly, the serum PON1 arylesterase activity of young and healthy adults increased significantly after eating 3 eggs per day for 4 weeks. In addition, with egg intake, the increase in large HDL particle concentration is consistent with the increase in lecithin cholesterol acyltransferase (LCAT) activity [18]. LCAT is a HDL-associated enzyme which is critical to promote the maturation of HDL by esterifying free cholesterol into cholesterol esters. Macrophages mobilization through HDL-C, known as cholesterol efflux, have shown in cohort studies that they can be used to predict the cause of CVD, independent of HDL-C level [144, 145]. It is worth noting that when adults with metabolic syndrome eat three eggs per day for 12 weeks, serum cholesterol excretion increases [145]. Generally speaking, in addition to increasing HDL-C, dietary cholesterol can also improve other indicators of HDL function. However, more investigation of the effect of dietary cholesterol on human HDL function is justified.

### 5.2. The Effects of Egg Components on Cholesterol Homeostasis

There are some nutrients in egg, such as ovomucin [146], sulfur-containing proteins [147–149], hydrolyzed proteins [106], and phospholipids [43], which may be considered bioactive and have their own functions. Those components may regulate cholesterol absorption and metabolism, which counteracts the adverse effects of high dietary cholesterol intake [150, 151]. However, which bioactive components would regulate the cholesterol metabolism and how they work remain to be studied. This argues the potential difference of effect of consuming pure cholesterol and the whole egg on the risk of CVD. Components other than cholesterol in egg may be bioactive and impact on cholesterol absorption, metabolism, transportation, and excretion.

#### 5.2.1. Protein and Amino Acids in Eggs

Eggs contain proteins that provide all essential amino acids for human nutrition. Individual amino acids such as glycine (Gly), glutamate (Glu), methionine (Met), and cysteine (Cys) in eggs play roles in regulating cholesterol metabolism. Met can participate in the formation of phospholipids, which are needed for the formation of lipoproteins. Glu, Cys, and Gly are used to synthesize glutathione, an activator of CYP7A1, which is critical for bile acid synthesis. In addition, Gly and Cys are involved in the formation of taurine, which is conjugated to bile acids [152].

Egg ovomucin attenuates hypercholesterolemia in rats and inhibits cholesterol absorption in Caco-2 cells [153]. The interplay of the cholesterol-containing micelles and the small intestinal epithelial cells inhibits the absorption of cholesterol and may be part of the cholesterol-lowering mechanism. Egg ovomucin may also inhibit the reabsorption of bile acids in the ileum, thereby lowering serum cholesterol levels [153]. Sulfur-containing proteins have been thought to increase HDL, decrease LDL, and attenuate atherosclerosis and metabolic syndrome. These proteins may affect lipid metabolism through modifications of transcriptional factors [147–149]. Manso et al. have shown in animal feeding and in vitro experiments that proteins from hydrolyzed total eggs can inhibit the absorption of cholesterol and may also affect the cholesterol metabolism in* vivo* [106]. A recent study suggested that the consumption of egg white in mice showed no significant changes in total cholesterol, HDL, LDL, or TG levels [154].

#### 5.2.2. Fatty Acids in Egg Yolk

Egg yolk contains abundant unsaturated fatty acids, which account for nearly two-thirds of the total lipids as shown in Table 1. These include n-3 PUFAs that are absorbed with cholesterol upon food intake. They may affect cholesterol uptake and plasma LDL-cholesterol level [155]. It seems that, compared with other cholesterol-rich foods such as meat, eggs have some unique characteristics. Fang et al. evaluated the effects of six fatty acids: palmitic acid, oleic acid, linoleic acid, arachidonic acid, EPA, and DHA on cholesterol uptake and transport in human enterocytes Caco-2 cells and on the mRNA expression levels of NPC1L1 and other proteins involved in cholesterol absorption. The results showed that EPA and DHA dose-dependently inhibited cholesterol uptake and transport in Caco-2 monolayer, which might be caused by downregulating NPC1L1 mRNA and protein levels [45].

An egg yolk contains nearly 331 *μ*g carotenoids lutein and zeaxanthin. Fernández-Robredo et al. have studied the influence of egg yolk and the protective effect of lutein supplement on the structural deterioration of retina in the apolipoprotein E deficient (apoE -/-) mice. The ingestion of egg yolk reduced the plasma TG level whereas total cholesterol remained constant, indicating its potential to reduce the risk of heart diseases [146].

#### 5.2.3. Phospholipids in Egg Yolk

Phospholipids are a major component of eggs as shown in Table 1. As a major component, phospholipids account for ~10% of the wet weight of egg yolk [42], which mainly contains PC (80.5%), PE (11.7%), lysoPC, SM, and some neutral lipids in minor quantities. Alfred Rampone reported that excess phospholipids could inhibit intestinal cholesterol uptake [156–158]. Then Rodgers and O'Connor hypothesized that lysoPC hydrolyzed from PC could lead to rapid uptake of cholesterol and attenuate the inhibitory effects of PC [159]. Microcapsules containing PC can reduce the absorption of cholesterol, esterification, and secretion. When half or more than half of the PC content in micelles is replaced by lysoPC, this inhibitory effect disappears [160]. Recently,* in vitro* studies using intestinal segments [161] or intestinal cell lines [74, 162] showed that phospholipids could alter the rates of micellar formation and diffusion to inhibit the intestinal uptake of cholesterol.* In vivo* studies have confirmed that native phospholipids exert an inhibitory effect on intestinal cholesterol absorption [61, 97, 163–166]. Recently, Fang et al. confirmed that the phospholipids PC and SM from egg yolk, which are considered as functional food ingredients, significantly inhibit the intestinal absorption of cholesterol in the Caco-2 monolayer [44]. Therefore, consuming eggs may inhibit the intestinal absorption of cholesterol via the phospholipids in eggs. A fundamental question is how this biological effect is regulated. Three possible mechanisms may be responsible for the inhibitory effect of excessive phospholipids on the intestinal absorption of cholesterol.

Firstly, the excessive intake of phospholipids interferes with its hydrolysis in micelles, which can promote cholesterol absorption [167]. Recent evidence showed the key role of phospholipase A2 (PLA2), a pancreatic secreted enzyme, in cholesterol absorption. The phospholipid composition in micelles and the effect of PLA2 on lipid uptake and metabolism have been demonstrated in human intestinal caco-2 cells [161]. The inhibitory effects of phospholipids on cholesterol absorption can be attenuated when PLA2 is added. Anti-PLA2 antibody has been shown to completely inhibit the action of the extracts from pig pancreas and inhibit the absorption of cholesterol optimization in Caco-2 cells [160]. PLA2 inhibitors (FPL67047XX) also have been shown to block cholesterol absorption [168]. All these indicate that small intestinal absorption of micellar cholesterol depends on the hydrolysis of phospholipids in micelles, and inhibition of the hydrolysis of phospholipids directly interferes with cholesterol intake. Therefore, elevated consumption of phospholipids may hinder or delay phospholipid hydrolysis in normal micelles and affect downstream cholesterol absorption.

The second kind of inference mechanism is that excess phospholipids affect the physical and chemical properties of micelles containing cholesterol (such as their size, composition, and biological properties). The phospholipids can cause cholesterol molecules from micellar phase into a lamellar (blister phase) which is difficult to be absorbed [169]. After chyme enters the duodenum from the stomach, the bile acids and lipids are present in different physical states (simple micelles, mixed micelles, and vesicles). The process of lipid hydrolysis by pancreatic enzymes, the accumulation of hydrolytic products, and the action of bile acids would transform the vesicles' shape and properties, which is necessary for lipid absorption. It can be speculated that excessive phospholipids may hinder the vesicle transformation, and phospholipids from different dietary sources can change the solubility of micelle, thereby preventing cholesterol to be absorbed by the small intestinal mucosa [169]. A study by Martin Carey's laboratory found that the degree of unsaturation of fatty acyl chains in phospholipids regulates the distribution of PC in micelles and the vesicles [170, 171]. The cholesterol solubility in the system of SM or saturated 2-palmitoylcholine is extremely poor [74]. Compared with eggs, the SM in milk, which contains fatty acids with higher saturation and longer chain length, can inhibit the absorption of cholesterol more effectively [98]. This indicates that the intestinal lumen phospholipid content and types will affect the physicochemical properties of intestinal micromicelles and, in turn, the absorption and metabolism of cholesterol.

The third inference mechanism involves the direct effect of phospholipids on cholesterol absorption in small intestine epithelial cells. It can be speculated that phospholipids in the intestinal lumen may affect cell membrane properties or directly affect intracellular cholesterol transport, thereby affecting intestinal cholesterol absorption. Fang et al. recently stated that egg yolk PC and SM could dose-dependently inhibit cholesterol uptake and transport in Caco-2 monolayer by regulating some intestinal cholesterol transport proteins and transcriptional factors [44]. SM downregulate the NPC1L1 expression levels in Caco-2 cells. Although few data could support this inference mechanism, it is well known that the biological function of the cell membrane highly depends on the phospholipid components and the interplay of the intramural cholesterol [172]. In addition, membrane protein activity can be directly influenced by its interaction with the membrane [173]. Therefore, dietary phospholipids may directly affect the uptake of cholesterol in the intestinal epithelium.

On the other hand, recent studies of gut microbiota show a different story. PC, like carnitine from red meat, is converted by intestinal bacteria into trimethylamine. Trimethylamine is oxidized in the liver to trimethylamine N-oxide (TMAO), and TMAO increases in a dose-dependent manner with egg consumption [174]. TMAO causes atherosclerosis in animal models [175] and in human subjects accelerates decline of impaired renal function [176] and increases cardiovascular risk [176, 177].

#### 5.2.4. Other Factors

Besides the common macronutrients, eggs are rich in glycine betaine, mercaptan, sialic acid, vitamins, and minerals; they might directly affect human body blood vessel functions or cholesterol metabolism by regulating activities of related enzymes or indirectly affect blood lipid level by regulating protein, fat, and carbohydrate metabolism. For example, Cys and glutathione may help to remove free radicals. Egg contains vitamins A, E, B2, and B6 and vitamin B12, which are able to resist LDL oxidation, lower homocysteine accumulation, dilate blood vessels, and prevent the happening of the angina pectoris, atherosclerosis, and myocardial infarction [85]. There are more than 40 kinds of trace elements in egg. They may play a vital role in the structure and function of cell membrane, lipid metabolism and stability, the contraction diastole heart, regulating blood pressure and blood coagulation and catalytisis, and inhibiting free radicals [85]. The ratio of LDL and HDL in egg is the same as that in human blood and LDL maintains pattern conversion. Despite the existence of cholesterol in the egg yolk, its impact on CVD may be different from the cholesterol in other food sources such as meat. In addition, when cholesterol is increased from food, the body makes less in healthy individuals. When the body's daily intake of cholesterol reaches more than 2 g, the absorption rate will gradually decrease, the synthesis of cholesterol in the body is reduced, and the neutral cholesterol content in the stool increases. If the cholesterol intake is low, cholesterol synthesis will increase to maintain a constant value [178].

## 6. The Relationship between Egg Intake and CVD Risks

### 6.1. Half-Century Debate of the Relationship between Dietary Cholesterol Intake and CVD Risks

Total body cholesterol is balanced via absorption from diet, biosynthesis in the body, and excretion out of the body [122]. In the 20th century, the dietary cholesterol and heart diseases hypothesis was proposed by the famous Framingham Heart Study, which reported the relationship between blood cholesterol and heart disease [3, 4]. This diet-heart disease hypothesis states that there is a causal relationship between dietary saturated fat and cholesterol intake and the serum cholesterol level, as a result increasing the risk of CVD [82]. According to this hypothesis, limiting the dietary cholesterol intake was advocated by animal studies [8, 179], observational studies [8, 12, 180, 181], and some dietary guidelines [8, 182]. In 1968, the AHA recommended that the dietary cholesterol consumption should be no more than 300 mg/day and emphasized no more than 3 egg yolks should be consumed per week [8].

Weggemans et al. [180] conducted meta-analysis of the epidemiologic studies to determine the relationship between dietary cholesterol intake and serum cholesterol level. Seventeen studies taken between 1979 and 1999 are included in this analysis. Fifteen studies observed an increase of total serum cholesterol level, 14 studies observed an increase of serum LDL, and 8 studies observed an increase of serum HDL, with the increasing of dietary cholesterol intake. They found that dietary cholesterol intake would increase the ratio of total to HDL cholesterol level, and with the addition of 100 mg dietary cholesterol intake per day, this ratio would increase by 0.020 units. Although both serum total and HDL cholesterol were increased, the increase of the ratio of total to HDL cholesterol indicates an overall adverse effect of dietary cholesterol intake on the serum cholesterol profile, which supports the diet-heart disease hypothesis [180]. Also, serum cholesterol predictive equations proposed by Keys (1965) and Hegsted (1993) included dietary cholesterol intake as a positive factor in addition to dietary saturated fatty acid intake [183, 184].

The average intake of dietary cholesterol in US adults ranges from 200 to 350 mg/day due to sex and age differences [9], which is quite close to the recommended consumption limitation by AHA [8]. Daily intake of eggs and egg products accounts for a quarter of daily cholesterol intake in US children and adults [10, 11]. Therefore, people tend to limit their consumption of eggs to decrease the cholesterol intake, in order to protect themselves from CVD. However, the controversy does not stop here, later studies do not support this hypothesis, and potential beneficial effects of egg on CVD have been reported [12–23, 150, 151, 185]. A prospective study with over 117,000 adults and 14-year follow-up demonstrated that there were no differences of CVD risks between participants that consumed one egg per week and those who consumed one egg per day [12]. Studies also found that eating large amount of eggs (10 eggs/day) does not significantly influence the serum cholesterol level comparing to the low intake (1 egg/day), and some even found there is a negative correlation [185]. Besides, egg consumption is not related to the incidence of CVD [15, 47, 186]. Whether the effect of egg yolk is worse than other dietary cholesterol resources on serum cholesterol level is still controversial [150, 151]. Therefore, some researchers recommended consuming egg to healthy people unless they are allergic or intolerant to egg, based on their findings that eggs are good resources for other nutrients. With increasing evidences, AHA eliminated the restriction of egg consumption in 2002, and the dietary restriction of cholesterol for healthy population in 2013 [24]. Eventually, in 2015, DGAC also eliminated this restriction from the latest version of dietary guideline [25]. However, comparing with other types of diet such as the Mediterranean one, Western diet in USA contains much more risk factors of CVD other than dietary cholesterol intake. Therefore, the adverse effect of dietary cholesterol might be shadowed by other risk factors [187]. Also noted is that the marginal relationship between dietary cholesterol intake and CVD risks is reported only in healthy people. Many studied reported doubling or even 5-fold increase of coronary disease risk as a result of an egg per day in diabetic participants [12, 13, 126].

### 6.2. Effect of Egg Intake on Blood Cholesterol and CVD in Animal Studies

Studies in animal models show that the impact of egg intake on blood cholesterol is minor. Fernández-Robredo gavaged apoE^−/−^ mice, a hypercholesterolemia and CVD model, with two egg yolks per day. The supplement of egg yolk in this amount did not increase the serum cholesterol; instead, the serum TG and lipid peroxidation were decreased [146]. Yang's study in Sprague-Dawley (SD) rats fed egg-enriched and high cholesterol diet observed decreased serum total cholesterol and increased HDL [188]. Egg-enriched diet increased the fecal excretion of sterols and bile acids, enhanced the HDL-reverse cholesterol transport and cholesterol hydroxylation indicated by elevated expression levels of CYP7A1, LCAT, and ACAT, and decreased the cholesterol biosynthesis in the presence of reduced expression of hepatic HMG-CoA reductase [188]. They speculated that some compounds such as phospholipids and certain protein could decrease the absorption of cholesterol in the intestine [146, 153].

Jiang et al. infused SD rats through lymph cannula with the lipid emulsion containing ^14^C-cholesterol and PC from different sources (egg PC, hydrogenated egg PC, and soy PC) [97]. The egg PC dramatically decreased the absorption of cholesterol, comparing to soy PC. This difference could be explained by the higher saturation of acyl groups in egg PC in comparison with that from soy [97]. This result indicates that the egg PC may also inhibit the intestinal absorption of cholesterol, a conclusion that is consistent with that of Rampone [156]. Other than PC, SM has also been discovered to inhibit the intestinal absorption of cholesterol in a dose-dependent manner, and this effect is achieved by decreasing the cholesterol monomers' thermodynamic activity [74, 189, 190]. Noh and Koo compared the inhibitory effects of SM from different sources, milk and egg, on cholesterol absorption, and found that milk SM had a stronger inhibitory effect probably due to higher saturation degree and longer chain of fatty acyl group in SM [98]. Feng found that ceramide, the hydrolytic product of SM, had a stronger inhibitory effect on cholesterol uptake in intestinal cells [191]. The possible mechanisms of inhibitory effect of phospholipids are discussed in Section 5.2.

Nagaoka investigated the effect of ovomucin, a major protein in egg, on the cholesterol metabolism both in vivo (Wistar rats) and in vitro (Caco-2 cells, a small intestinal enterocyte model) [153, 192]. They found that feeding rats with ovomucin decreased the serum total cholesterol level and the total lipids level in the liver [153].* In vivo* study showed that the fecal excretion of bile acids and cholesterol was significantly increased in rats fed ovomucin in comparison with those fed casein [153]. Other investigations on the effect of total egg white protein (EWP) on cholesterol metabolism also showed some beneficial outcomes. Matsuoka fed SD rats with EWP and observed the decreased cholesterol level in the serum, liver, and intestinal mucosa [193]. Both the fecal excretions of sterols and bile acids were increased, and the cholesterol micellar formation was interfered, which probably was due to the reduced cholesterol absorption in rats fed EWP [193]. In another study, spontaneously hypertensive rats were fed the pepsin hydrolyzed egg white (hEWP) and showed that the serum total cholesterol was decreased without changing HDL [163]. A research in human females with moderate hypercholesterolemia also observed the beneficial effects of EWP, which included a decrease of serum total cholesterol and increase of HDL compared to the group fed with tofu or cheese as the protein resource [162].

All those results in animals showed the potential beneficial effects of egg on blood cholesterol level through decreasing the absorption and increasing excretion, and several functional compounds were proposed. However, more experiments through feeding animals with egg yolk and monitoring CVD related indictors other than blood cholesterol are required to determine the effect of consuming eggs on CVD risks in animals. Also, other functional compounds may not have been screened out yet, and the molecular mechanisms of function remain to be studied in depth.

### 6.3. Effect of Egg Intake on Blood Cholesterol and CVD in Human Studies

The effects of egg intake on blood cholesterol and CVD have been discussed in several meta-analysis studies using research data collected over 60 years [15, 21–23, 180, 194]. Large epidemiological works have been conducted to investigate the effect of egg intake on blood cholesterol levels and risk of CVD in children [123], young people [16, 20, 47, 124], women [12–14, 125, 195, 196], men [12–14, 125, 195–199], and older adults [12–14, 125, 195–200]. Some have shown that egg consumption did influence the blood cholesterol level but did not increase the risk of CVD in healthy people. Meanwhile, other studies reported that high dietary cholesterol intake due to egg consumption is a risk factor for CVD and diabetes [180, 194, 197, 199, 201–204]. The results of epidemiological studies and human intervention studies on the relationship of dietary egg intake and CVD risks are summarized in Table 3. Even though AHA and DGAC have removed the restriction of dietary cholesterol for healthy people in USA, there still are different conclusions due to differences in race, genetic makeup, physical fitness, and especially physiological status.

Among the 19 prospective studies investigating the effect of dietary egg intake on CVD risks, 6 studies reported positive correlation between egg consumption and different types of CVD incidents or mortality in healthy people [126, 195, 197, 205, 208, 209]. Pang (2017) reported the positive correlation with total cholesterol [211], and Spence (2012) reported the positive correlation with plaque area [212]. However, other studies (11 out of 19) reported no difference on the CVD risks affected by the amount of egg intake. The adverse effect of egg consumption is observed in population with high risk of CVD, including people with diabetes or hypercholesterolemia, and who are sensitive to dietary cholesterol [12, 13, 126, 131, 210, 213]. Diabetic populations are in the high risk of CVD with two to four folds higher than healthy people. These studies also showed that diabetic people are more vulnerable to CVD after egg consumption [13, 208], with a doubling of coronary risk with an egg per day in US population [12, 13], and 5-fold risk in Greece population [213]. Meanwhile, some studies found that high egg consumption increased the risk of gestational diabetes mellitus [214], insulin resistance [215], and the risk of diabetes [202–204]. Therefore, the effect of egg consumption on CVD might be mediated by diabetes.

Almost all human intervention studies showed the serum LDL and HDL cholesterol levels increased in high egg consuming groups (1 to 3 eggs per day comparing to no egg or with egg substitute), while the ratio of serum LDL to HDL (LDL/HDL) is unchanged (Table 3). Most of these papers concluded that egg consumption is not a risk factor for CVD, based on the fact that the LDL/HDL ratio is unchanged because this ratio is thought to be a stronger risk factor for CVD. However, serum LDL level alone should still be considered as a risk factor for CVD. This is especially true for those people whose blood cholesterol level is more sensitive to dietary cholesterol consumption. There are good reasons for the recommendation that persons at risk of vascular disease limit cholesterol to 200 mg/day [186]. The very high cholesterol content of egg yolk (237 mg in a 65-gram egg) is a problem in itself, and even one large egg yolk exceeds that limit. Other studies reported the high cholesterol and high lipid diet could induce the inflammation in plasma, which is thought to contribute to atherosclerosis [216], and the susceptibility of LDL to be oxidized could be increased by dietary cholesterol [217].

## 7. Outlook

Interestingly, current studies have tended to show that the consumption of eggs is not a risk factor of CVD in healthy people. However, people who are at high risk of CVD such as those with diabetes or hypertension need to have caution with dietary cholesterol intake, especially egg intake. Also, some people seem to be more sensitive to dietary cholesterol whose blood cholesterol level is highly correlated to dietary intake. Therefore, even though the recommendation of restricting cholesterol and egg consumption in AHA and DGAC has been eliminated, we still need to have caution with them based on the physiological status of people. On the other hand, the studies on the egg components impacting CVD risk showed that some egg components have potential protective effects on CVD, while others may have adverse effects. Due to the lack of complete data, the components of eggs that regulate cholesterol absorption and metabolism have not been extensively studied systematically. To solve the mystery of the relationship between egg cholesterol and blood cholesterol, it is essential to understand intestinal absorption of cholesterol from eggs and study the effect of cholesterol in eggs, and nutrients and cholesterol interactions in eggs. Also, the function of gut microbiota needs to be taken into consideration as well. Overall, in order to strengthen the basic research of egg functional components, understanding of the nutritional value of eggs can provide theoretical data for reasonable determination of the intake of eggs.

## Figures and Tables

**Table 1:** Nutrient values of a medium-size boiled egg, whole milk with added vitamin D, and boiled manufacturing beef^a^.

**Category**		**Boiled egg**		**Whole milk with added vitamin D**	**Boiled manufacturing beef**

**Nutrient**	**Unit**	**1 large** **(50.0 g)**	**1Value** **(100 g)**	**1Value** **(100 g)**	**1Value** **(100 g)**

**Proximates**					
Energy	kcal	78	155	61	126
Water	g	37.31	74.62	88.13	73.1
Protein	g	6.29	12.58	3.15	24.21
Total lipid (fat)	g	5.3	10.61	3.25	3.26
Carbohydrate, by difference	g	0.56	1.12	4.8	0
Fiber, total dietary	g	0	0	0	0
Sugars, total	g	0.56	1.12	5.05	0

**Minerals**					
Calcium, Ca	mg	25	50	113	6
Iron, Fe	mg	0.59	1.19	0.03	1.78
Magnesium, Mg	mg	5	10	10	16
Phosphorus, P	mg	86	172	84	129
Potassium, K	mg	63	126	132	183
Sodium, Na	mg	62	124	43	32
Zinc, Zn	mg	0.53	1.05	0.37	5.02

**Vitamins**					
Vitamin C, total ascorbic acid	mg	0	0	0	0
Thiamin	mg	0.033	0.066	0.046	0.042
Riboflavin	mg	0.257	0.513	0.169	0.096
Niacin	mg	0.032	0.064	0.089	1.759
Vitamin B_6_	mg	0.06	0.121	0.036	0.16
Folate, DFE	*μ*g	22	44	5	0
Vitamin B_12_	*μ*g	0.56	1.11	0.45	1.02
Vitamin A, RAE	*μ*g	74	149	46	8
Vitamin A, IU	IU	260	520	162	27
Vitamin E (*α*-tocopherol)	mg	0.52	1.03	0.07	0.57
Vitamin D (D_2_ + D_3_)	*μ*g	1.1	2.2	1.3	0.1
Vitamin D	IU	44	87	51	5
Vitamin K (phylloquinone)	*μ*g	0.1	0.3	0.3	0

**Lipids**					
SFAs^b^	g	1.633	3.267	1.865	1.154
MUFAs^b^	g	2.038	4.077	0.812	0.897
PUFAs^b^	g	0.707	1.414	0.195	0.246
Trans fatty acids	g	0	0	0	0.078
Cholesterol	mg	186	373	10	67

^a^Nutrient values and weights are for edible portion; ^b^SFAs: saturated fatty acids, MUFAs: monounsaturated fatty acids, and PUFAs: polyunsaturated fatty acids.

**Table 2:** Possible factors influencing intestinal cholesterol absorption^a,b^.

**Factors**	**Influencing** ^**c**^	**Research method**	**References**
**(A) Dietary Factors**			
↑ Cholesterol	(-)	Mouse feeding studies	Duan 2004 [64]
Fat			
↑ Stearic Acid	↓	Rat feeding studies	Kelley 1978 [65]; Vahouny 1988 [66]
↑ MUFAs	↓	African green monkey feeding studies	Johnson 1985 [67]
↑ *ω*-3 PUFAs	↓	African green monkey feeding studies Caco-2 cells	Johnson 1985 [67] Fang 2018 [44]
↑ Fish Oil	↓	Rat feeding studies	Chen 1987 [68]
↑ Cellulose	↓	Hamster feeding studies	Turley 1994 [69]
↑ Phytosterols	↓	Human intervention (see table in reference)	Nguyen 1999 [70]
↑ Soluble Fibers	↓	Rat and hamster feeding studies	Feldman 1979 [71]; Schneider 2000 [72]
↑ Ezetimibe	↓	Human and hamster feeding studies	Rosenblum 1998 [73]; Davis 2004 [53]
↑ Sphingomyelin	↓	Mouse feeding studies	Eckhardt 2002 [74]
**(B) Bile acid factor**			
↓ Bile acid output	↓	Cyp7a1(-/-) mice	Schwarz 2001 [55]
↓ Bile acid/salt pool size	↓	Cyp7a1(-/-) mice	Schwarz 2001 [55]
↓ Bile acid phospholipid output	↓	Abcb4(-/-) mice	Wang 1998 [54]
↑ Bile acid cholesterol output	↑	Various mouse strains	Wang 2001 [60]
↑ Cholesterol content in bile salts	↑	Various mouse strains	Wang 2001 [60]
↑ Hydrophobic bile salt	↑	Mouse feeding studies	Wang 2003 [57]
↑ Hydrophilic bile salt	↓	Mouse feeding studies	Wang 2003 [57]
**(C) Genetic factors**			
↓ ACAT2	↓	Inhibitor in mice Acat2(-/-) mice	Buhman 2000 [75]; Clark 1984 [76]
↓ HMG-CoA R	↓	Inhibitor in human and mice	Hajri 1995 [77]; Vanhanen 1992 [78]
↓ ABCA1^d^	↓ ↑	Abca1 (-/-) mice	McNeish 2000 [79]; Drobnik 2001 [80]
↓ ABCG5 and ABCG8	↑	Abcg5/g8(-/-)mice	Yu 2002 [81]
↓ NPC1L1	↓	Npc1/1(-/-) mice Inhibitor in mice	Altmann 2004 [82]; Davis 2004 [53]
↓ SR-BI^d^	(-)	Sr-b1 (-/-) mice	Mardones 2001[83];
↑ SR-BI	↑	Overexpression in CHO cells	Altmann 2002 [84];
↑ SR-BI	↓	Hepatic overexpression in mice	Sehayek 1998 [85]
↓ Caveolin1	(-)	Cav1 (-/-) mice	Valasek 2005 [86]
↓ MTP	↓	Inhibitor in human	Samaha 2008 [87]; Cuchel 2007 [88]
↓ APO-B48	↓	ApoB48 (-/-) mice Apo-B100-only mice	Young 1995 [89]; Wang 2005 [90]
↑ LXRs	↓	Agonist in human	Repa 2000 [91]
↑ FXR	↓	Agonist in human	Repa 2000 [91]
↑ RXR*α*	↓	Agonist in mice	Repa 2000 [91]
↑ PPAR*α*	↓	Ppar*α* (-/-) and agonist in mice	Knight 2003 [92]
↑ PPAR*δ*	↓	Agonist in mice	Van Der Veen 2005 [92]
**(D) Intestinal lumen factor**			
↑ Small intestine transit time	↑	Cck-1 receptor (-/-) mice	Wang 2004 [59]
↑ Gastric emptying time	↑	Various of mouse strains	Kirby 2004 [93]
↓ MUC1	↓	Muc1 (-/-) mice	Wang 2004 [59]
↓ CEL^d^	(-) ↓	Cel (-/-) mice Cel (-/-) mice	Kirby 2002 [94] Weng 1999 [95]
↓ PTL	↓	Ptl (-/-) mice	Huggins 2003 [96]

^a^ Table 2 is modified from Wang 2005 [90] with supplement. ^b^Abbreviations: MUFAs, monounsaturated fatty acids; PUFAs, polyunsaturated fatty acids; CYP7a1, cholesterol 7*α*-hydroxylase; ABC, ATP-binding cassette (transporter); ACAT2, acyl-CoA:cholesterol acyltransferase, isoform 2; HMG-CoA R, HMG-CoA reductase; NPC1L1, Niemann-Pick C1-Like 1; SR-BI, scavenger receptor class B member I; MTP, microsomal triglyceride transfer protein; APO, apolipoprotein; LXR, liver X receptor; FXR, farnesoid X receptor; RXR, retinoid X receptor; PPAR, peroxisomal proliferator activated receptor; CCK, cholecystokinin; MUC, mucin gene; CEL, carboxyl ester lipase; PTL, pancreatic triglyceride lipase; ^c^↑ increase, ↓ decrease, (-) no influence. ^d^Contradictory result from different research groups.

**Table:** (a) Prospective studies

**Reference**	**Participants**		**Age**	**Follow-up (years)**	**Outcome**	**Result** ^**b**^
	**Male**	**Female**				
Bernstein 2011 [178]	43,150	84,010	30-75	26	Incident stroke	(-)
Burke 2007 [205]	256	258	15-88	14	CHD, mortality	↑
Dawber 1982 [185]	912		30-59	24	Incident CHD and blood cholesterol level	(-)
Djoussé 2008 [197]	21,327	0	40-85	20	Incident MI and stroke	(-)
					Mortality	↑
Goldberg 2014 [206]	572	857	57-75	11	Incident stroke	(-)
					Carotid atherosclerosis	↓
Haring 2014 [207]	12,066		45-64	22	Incident CHD	(-)
Houston 2011 [208]	864	1077	70-79	9	Incident CVD	↑ especially in diabetic people
Hu 1999 [12]	37,851	80,082	34-75	14	Incident stroke and CHD	(-) while in diabetic people may have ↑ effect
Mann 1997 [209]	4,102	6,700	16-79	13.3	Ischemic heart disease mortality	↑
Nakamura 2004 [195]	5,186	4,077	30-70	14	Stroke and CHD mortality	↑ in women
Nakamura 2006 [14]	43,319	47,416	40-69	10.2	Incident CHD	(-)
Qureshi 2006 [13]	3,756	5,978	25-74	15.9	All stroke, CAD	(-) while in diabetic people may have ↑ effect
Sauvaget 2003 [210]	15,350	24,999	34-103	16	Stroke mortality	(-)
Scrafford 2011 [196]	14,946		>17	8.8	CHD and Stroke mortality	(-)
Zazpe 2011 [45]	6,170	8,015	20-90	5.8	Incident CVD	(-)
Voutilainen 2013 [198]	1,019	0	51.9 (Mean)	18.8	Carotid atherosclerosis, incident MI	(-)
Pang 2017 [211]	8,131	8,463	>60	N/A	Serum LDL and total cholesterol	↑
Spence 2012 [212]	669	593	46-77	N/A	Carotid plaque area	↑
Trichopoulou 2006 [126]	424	589	50-80 (Adult diabetics)	4.5 (mean)	Mortality	↑

**Table:** (b) Human intervention

**Reference**	**Participants**		**Age**	**Intervention time (weeks)**	**Intervention method**	**Outcome**	**Result** ^**a**^
	**Male**	**Female**					
Missimer 2017 [124]	24	26	18-30	11^c^	2 eggs/day vs. oatmeal	Serum LDL and HDL	↑
						Serum LDL/HDL	(-)
						Serum ghrelin	↑ satiety

Lemos 2018 [20]	16	14	18-20	13	3 eggs/day vs. choline bitartrate supplement	Serum LDL and HDL	↑
						Serum LDL/HDL	(-)
						SREBPs and HMG-CoA reductase level	↓ cholesterol biosynthesis

Herron 2002 [125]	0	51	18-49 (pre-menopausal)	11^c^	1 egg/day vs. 0 egg/day	Serum LDL and HDL	↑
						Serum LDL/HDL	(-)
						CETP level	↑ reverse cholesterol transport

Herron 2003 [126]	40	0	18-57	11^c^	1 egg/day vs. 0 egg/day	Serum LDL and HDL	↑
						Serum LDL/HDL	↑ only in hyper-responders^d^
						CETP, LCAT level	↑ reverse cholesterol transport

Mutungi 2008 [199]	28	0	40-70 (overweight/obese)	12	CRD: 3 eggs/day vs. SUB	Serum LDL/HDL	(-)
						Serum HDL	↑

Greene 2005 [200]	13	29	>60	11^c^	3 eggs/day vs. SUB	Serum LDL and HDL	↑
						Serum LDL/HDL	(-)

Ballesteros 2004 [123]	25	29	8-12	11^c^	2 eggs/day vs. SUB	Serum LDL/HDL	(-)

Knopp 2003 [132]	78	119	43-67	4	0, 2 and 4 eggs/day	Serum LDL and HDL	↑

Knopp 1997 [131]	86	45	41-68 (HC or HL)	12	2 eggs/day vs. SUB	Serum LDL	↑ in HC
						Serum HDL	↑ in both HL and HC

^a^Abbreviations: CHD, coronary heart disease; CVD, cardiovascular disease; MI, myocardial infarction; LDL, low-density lipoprotein; HDL, high-density lipoprotein; SREBP, sterol regulatory element-binding protein; CETP, cholesteryl ester transfer protein; LCAT, lecithin-cholesterol acyltransferase; CRD, Carbohydrate-restricted diets; SUB, cholesterol-free, fat-free egg substitute; HC, hypercholesterolemia; HL, hyperlipidemia; ^b^↑ increase, ↓ decrease, (-) no influence. ^c^Intervention time contain a 3-weeks washout time within the intervention period; ^d^hyperresponders: increase in total cholesterol of ≥0.06 mmol/L for each additional 100 mg of dietary cholesterol consumed.
